# Health Literacy and Cognitive Disorders in Diabetic Patients

**DOI:** 10.3390/jcm14092972

**Published:** 2025-04-25

**Authors:** Magdalena Florek-Łuszczki, Piotr Lutomski, Agnieszka Strzelecka, Jarogniew J. Luszczki, Piotr Dziemidok

**Affiliations:** 1 Department of Medical Anthropology, Institute of Rural Health, 20-090 Lublin, Poland; magdalena.florek@wp.pl (M.F.-Ł.); lutomski.piotr@imw.lublin.pl (P.L.); 2 Collegium Medicum, The Jan Kochanowski University, 25-516 Kielce, Poland; agnieszka.strzelecka@ujk.edu.pl; 3 Department of Occupational Medicine, Medical University of Lublin, 20-090 Lublin, Poland; 4 Department of Diabetes, Institute of Rural Health, 20-090 Lublin, Poland; dziemidok.piotr@imw.lublin.pl

**Keywords:** health literacy, cognitive impairment, diabetes

## Abstract

**Background/Objectives:** Diabetes is a chronic metabolic disease affecting over 500 million adults worldwide, which is over 10% of the world’s population. Diabetes is associated with a high risk of complications, including cognitive impairment of varying severity. Effective treatment of diabetes requires the patients not only to follow medical recommendations, but also to have appropriate health literacy (HL). The aim of the study was to determine the level of health literacy in diabetes patients, taking into account their cognitive functions. **Methods**: the study design consists of an anonymous survey involving 312 patients with type 1 and 2 diabetes, treated at the Diabetology Clinic of the Institute of Rural Health in Lublin, Poland. The survey was based on two standardized research tools, the 47-item European Health Literacy Questionnaire (EU-HLS-Q47) and the Mini-Mental State Examination (MMSE), and an original questionnaire focusing on the patients’ health situation, metric questions, questions about self-assessment of knowledge, and educational needs. **Results**: The EU-HLS-Q47 and MMSE showed that diabetic patients mostly presented a sufficient level of health literacy. A limited level of health literacy was presented by 36.86% of the examined diabetic patients. A statistically significant relationship between the length of diabetes (in years) and the General Health Literacy, Health Care, and Health Promotion Indices was reported. The MMSE test showed that every third patient with diabetes had cognitive disorders of varying intensity. **Conclusions**: Patients with diabetes and their family members require coordinated care and targeted therapeutic education to prepare them for self-care and self-control so as to reduce the risk of complications.

## 1. Introduction

Diabetes is a chronic metabolic disease affecting over 500 million adults worldwide, which is over 10% of the world’s population. Diabetes has a significant impact on the patients’ quality of life, their ability to work, their mental state and creates a significant burden on healthcare systems [1].

Diabetes, as a chronic disease, has a significant negative impact on the functioning of people in both physical and psycho-social spheres. Untreated or improperly treated, diabetes can result in ischemic heart disease, hypertension, ischemia of the lower limbs, or stroke [2]. The fact of having diabetes is correlated with the risk of other depressive symptoms, the occurrence of which is often associated with a high BMI, diabetic neuropathy, as well as a lack of professional activity and the need to use assistance in everyday functioning. An increased BMI and depression significantly reduce the quality of life of people with type 2 diabetes, especially those of senior age [3,4]. Of particular concern is the ever-increasing number of people with type 2 diabetes, which is strongly linked to improper high caloric diet, aging, and low physical activity [5,6].

Effective treatment of diabetes requires the patients not only to follow medical recommendations, including taking medications, following a diet, and leading a healthy lifestyle in accordance with the recommendations of healthcare providers [7], but also having the appropriate health literacy (HL). These competences are defined as the ability to obtain, understand, and use health information in order to make informed decisions about one’s own health. These skills include monitoring blood glucose levels, appropriate diet management, regular physical activity, and systematic and correct use of medications. Strict control of these aspects is crucial in preventing diabetes complications, such as retinopathy, nephropathy, or neuropathy [8,9,10].

The consequences of insufficient health knowledge among patients with diabetes are often unhealthy behaviors, low levels of satisfaction and quality of life, and in some cases, increased mortality [11]. People with limited health literacy have problems with understanding oral and written information provided by doctors, nurses, and information about medications. They also tend to have little knowledge about the organization of health care and are less likely to use preventive examinations and available medical services [12,13,14].

A high level of health literacy, especially in terms of obtaining information about health, is essential to protect against threats resulting from the use of unreliable information about health care or treatment methods [15], as well as to strengthen the patient’s position, knowledge, and skills in the field of self-control of health and self-treatment [16]. The effectiveness of self-management of diabetes also depends to a large extent on the patient’s mental state, which can significantly affect the ability to make decisions related to treatment. Mental disorders, such as depression, anxiety or stress, and cognitive disorders are common among people with diabetes and can lead to poorer glycemic control [17]. Central changes in the course of diabetes, both type 1 and type 2, include changes in the structure of brain tissue, electrophysiological abnormalities and disturbances in neurotransmission, as demonstrated by experimental and clinical studies [18]. All of these abnormalities can lead to cognitive decline, mainly due to complications of diabetes and extremely high or low glucose levels [19]. Therefore, it is important to conduct research for monitoring the level of the patients’ health literacy, which will be the basis for targeted actions aimed at its increase and resulting in the reduction of disease complications. Thus, the aim of the study was to determine the level of health literacy in diabetes patients, taking into account their cognitive functions.

## 2. Materials and Methods

### 2.1. Study Design

The research methodology employed a quantitative, descriptive, and correlational cross-sectional study design based on two standardized research tools—the 47-item European Health Literacy Questionnaire (HLS-EU-Q47) and the Mini-Mental State Examination (MMSE) test—and an original questionnaire.

### 2.2. Procedure

The study involved patients with type 1 and 2 diabetes, treated at the Diabetology Clinic of the Institute of Rural Health in Lublin, Poland. The selection of 312 patients for the study was purposeful and the study was conducted in the years 2020–2023 using the diagnostic survey method, and was anonymous. The inclusion criteria in this study were as follows: (1) age of the patients over 18 years; (2) staying in the Diabetology Clinic; (3) confirmed diagnosis of type 1 or 2 diabetes; (4) intellectual abilities enabling participation in the study; and (5) informed consent to participate in the study. In contrast, the exclusion criteria were based mainly on intellectual inabilities to participate in this study or diabetes not confirmed.

### 2.3. Population and Sample

According to data from the Central Statistical Office (GUS) and the National Health Fund (NFZ) in Poland, approx. 3.1 million people with diabetes have used public health care services in Poland in 2022. However, prognoses indicate that by 2030 this number may increase to 4.2 million. Considering the mentioned numbers of diabetic patients in Poland, the ideal sample size of surveyed patients with diabetes amounted to 271 people (at the 90% confidence level and margin of error of α = 0.05) (Qualtrics—Sample size calculator); thus, a total number of 312 surveyed patients in this study allowed to achieve representativeness for the population of diabetic patients in Poland.

The socio-demographic characteristics of the respondents are presented in Table 1. The percentage of patients, taking into account their gender, was similar, with a slight predominance of men, who constituted 56.4% of the total. The average age of the patients included in the study was 54.36 (SD = 15.89 years), the dominant age range was 51–70 years (51.9%), and every fourth patient was in the category of 31–50 years (25.3%). Almost 60% of the respondents were city residents. The level of education of the patients varied. The highest percentage was observed in patients with secondary or post-secondary education (40.1%) and vocational education (28.8%), while the lowest percentage was in patients with primary education (9.6%).

### 2.4. Instruments

The comprehensive health literacy survey tool—the 47-item European health literacy questionnaire (HLS-EU-Q47)—enables distinguishing health literacy between levels of education attainment of patients.

The Mini-Mental State Examination (MMSE) enables the detection of cognitive deficits and the assessment of their severity, especially in the elderly population and patients with chronic diseases. MMSE enables the assessment of aspects such as orientation, memory, attention, language skills, and executive functions. The use of this tool among patients with diabetes can help in the early detection of cognitive disorders [20,21]. For the MMSE scale, according to the adopted key, the following ranges were adopted: 30–27—normal result; 26–24—cognitive impairment without dementia; 23–19—mild dementia; 18–11—moderate dementia; 10–0—profound dementia.

The original questionnaire contained questions regarding the patients’ health situation, personal data questions, questions about self-assessment of knowledge, and educational needs.

### 2.5. Ethical Consideration

The study was conducted in accordance with the ethical principles of the Helsinki Declaration and received the approval of the Bioethics Committee of the Institute of Rural Health in Lublin (Approval Code: 5/2019; Approval Date: 25 April 2019). All patients who took part in the study gave their informed consent, were informed about the purpose of the study, as well as about the possibility of withdrawing from participation in the study at any stage.

### 2.6. Statistical Analysis

The internal consistency of the HLS was checked based on Cronbach’s alpha. Factor analysis was also performed for individual dimensions of the HLS tool. The Spearman’s rho correlation analysis was used to examine the relationship between individual ordinal variables and quantitative variables and individual dimensions of the HLS and MMSE questionnaires. The non-parametric Mann–Whitney U test was used to examine the difference in distributions of quantitative variables due to individual grouping variables (in case of lack of normality of the analyzed distributions). The χ^2^ test was used to determine the relationship between qualitative variables and individual dimensions of the HLS tool and the results of the MMSE test. For qualitative variables, the distribution and frequency were given, and for quantitative variables, the mean values and standard deviation. In all statistical tests, the level of significance was set at α = 0.05. Statistical analysis was performed using the software STATISTICA PL v. 13.3.

## 3. Results

All the patients who took part in the study had diagnosed diabetes. Of these, 59.62% were the patients with type 2 diabetics, while the rest (40.38%) were the patients with type 1 diabetes.

The internal consistency of the tool for measuring health literacy HLS-EU-Q47 in the Polish version, determined on the basis of Cronbach’s alpha, was 0.951 for the entire tool, and for its three subclasses, it was 0.890 for Health Care, 0.891 for Prevention of Disease, and 0.895 for Health Promotion, respectively. The highest mean values in the Health Care area were obtained for statement Q1.15 (which concerned the ease of calling an ambulance by the respondents in case of emergency) (3.60 ± 0.75) and the lowest values for Q1.1 (indicating the difficulty in finding information on the symptoms of the disease experienced by patients, i.e., diabetes) (1.47 ± 0.60). The highest mean values in the Prevention of Disease area were obtained for the statement Q1.21 (concerning understanding of the health warning covering behaviors such as smoking, low physical activity and drinking excessive amounts of alcohol) (3.51 ± 0.74) and the lowest for Q1.31 (concerning the decision on how the respondents can protect themselves from the disease based on information provided in the media) (2.97 ± 1.11). In the case of the Health Promotion subclass, the highest mean values were obtained for the statement Q1.42 (assessment of how the housing conditions of patients under observation help maintain health) (3.45 ± 0.78) and the lowest for Q1.35 (learn about political changes that may affect health) (2.94 ± 1.15) (Table 2).

More than half of patients (57.80%) struggled with diabetes complications. The average number of years of complications was 3.55 (SD = 5.709). For the patients with type 1 diabetes, the average was M = 3.15 (SD = 5.579), and for the patients with type 2 diabetes the average was M = 3.82 (SD = 5.795). Statistical analysis showed that the age of patients was a factor significantly differentiating patients, taking into account the type of diabetes and gender (Table 3).

The MMSE test showed that 67% of the examined patients with diabetes did not have cognitive impairment, but cognitive impairment without dementia affected 24.70% of patients; mild dementia—7.40%, moderate dementia—0.80%, and profound dementia only 0.30%. The average MMSE result for the entire sample was 27.12 (SD = 2.755). For patients with type 1 diabetes—27.20 (SD = 2.589), while for patients with type 2 diabetes—27.00 (SD = 2.865).

In order to assess the level of health literacy of the patients included in the study, we performed an analysis of the means and percentage distributions of the general health literacy index as well as individual dimensions of competences (such as health care, prevention of disease, and health promotion), assuming in accordance with the interpretational recommendations that the average score was within the range of >42–50 (excellent), >33–42 (sufficient), >25–33 (problematic), 0–25 (inadequate), together (problematic) and (inadequate) 0–33 (limited).

The obtained data showed that the patients included in the study presented a sufficient level of health literacy (Table 4). The general health literacy index was 36.05 ± 8.46. The average for the individual areas of health literacy did not differ significantly. Almost every third diabetic patient examined herein presented an excellent level of health literacy in the area of prevention of disease (30.13%), health promotion (29.49%), and every fourth in the area of health care (25.32%). In turn, a limited level of health literacy was presented by approximately 40% of the examined patients with diabetes, with the highest percentage of the examined people with this note referring to the area of health promotion (42.95%), for the area of health care it was 38.14%, and for the prevention of disease it was 38.78%.

Statistical analysis with the Mann–Whitney U Test was performed on the type of diabetes that the patients suffered from, as well as variables, such as the duration of the disease, the age of the patients, the general level of health literacy and its individual three areas, and the results of the MMSE test (Table 5). The analysis showed that only the age of the patients was statistically significant with respect to the type of diabetes (*p* < 0.001). The remaining parameters were not statistically significant with respect to the type of diabetes (Table 5).

In order to determine the relationship between the level of health literacy and independent variables, a correlation analysis was performed. The variable “education” was coded as an ordinal scale from the lowest to the highest level of education as follows: (1) primary, (2) vocational, (3) secondary or post-secondary, and (4) higher, which allowed for determining the relationship between the level of education and individual dimensions of the general health literacy Index and MMSE (Table 6). Based on the Spearman’s rho correlation analysis, a statistically significant relationship was demonstrated only between the level of education of the examined patients and the results of the MMSE test (*p* < 0.001)—the lower the level of education, the higher the share of patients with cognitive disorders or dementia. No statistically significant relationship was found between the level of education and individual dimensions of general health literacy, nor was there a statistically significant relationship between the level of MMSE values and individual dimensions of general health literacy (Table 6).

Additionally, a statistically significant relationship was found between the age of the patients and the results of the MMSE test (*p* < 0.001). It was found that the higher the age of the patients, the higher the share of patients with cognitive impairment or dementia. No statistically significant relationship was found between age and individual dimensions of general health literacy (Table 6).

Moreover, a statistically significant relationship was found between the duration of diabetes (in years) of the studied patients and the general health literacy index (*p* = 0.012), the health care index (*p* = 0.008) and the health promotion index (*p* = 0.004) (Table 6). It was found that the higher the number of years of diabetes, the higher the indicators of the tool and its subscales. No statistically significant relationship was found between the duration of the disease in the studied patients and the prevention of disease index or the MMSE test result (Table 6).

The χ^2^ test did not show a statistically significant relationship between the occurrence or lack of diabetes complications and individual dimensions of the HLS and MMSE.

## 4. Discussion

Diabetes, as a chronic disease, requires systematic and often long-term treatment. Compliance with therapeutic indications means preventing complications and effective control of diabetes, which is only possible with the active participation of patients in treatment plans, in accordance with the doctor’s recommendations [22]. Barriers to adherence to treatment may be related to patients’ financial problems, poor communication with the healthcare team, lack of family support, lack of knowledge, misconceptions, and limited health literacy [23]. Studies show that health literacy is one of the important factors influencing treatment adherence among patients with diabetes [24,25].

Due to the fact that research in the area of health literacy, including patients with diabetes, is not homogeneous in terms of the used tools (studies by different authors described 47-item, 16-item and 12-item tools), we have referenced only to the available publications.

The results showed that the patients participating in this study presented a sufficient level of health literacy, as indicated by the mean score of the obtained answers. The percentage of patients included in the study with regard to general HL at the level of excellent and sufficient in total was 63.14% (including excellent general HL—25.32% and sufficient general HL—37.82%). A more detailed analysis, taking into account the areas of health literacy, showed that every third examined patient with diabetes presented an excellent level of health literacy in the area of prevention of disease and health promotion, and every fourth in the area of health care. A limited level of health literacy was presented by 36.86% of the examined patients with diabetes, with the highest percentage of the examined people with this note referring to the area of health promotion, and lower for the areas of health care and prevention of disease; however, these differences were not statistically significant.

The obtained results showed that the level of health literacy of the examined patients with diabetes is higher than the general HL for the Polish society. The study conducted using the HLS-EU-Q47 questionnaire, as a part of international studies in Europe, showed that for the Polish population, limited general HL concerned 47.6% of people (problematic general HL—inadequate, including sufficient general HL (36.0%) and excellent general HL—16.5%) [26], and at the same time, it was similar to the result obtained in a study of patients with diabetes living in Norway, where, based on research using the HLS-12 questionnaire, it was found that on average 38% of patients with type 2 diabetes presented a limited level of health literacy [27].

In turn, the results of the study using HLS-EU-Q47, conducted among Spanish diabetic patients aged 50–75, showed that as many as 81.5% had a limited level of reading and writing skills [28]; however, it should be noted that the age limits of the study participants excluded the patients from younger age groups, which could, undoubtedly, significantly affect the obtained indicators.

An insufficient HL level was observed in 61.0% of the South Korean study participants [29], and about 46% of the Iranian residents surveyed [30], but, it should be noted that the two abovementioned studies concerned the general population, not diabetic patients. Significantly different results were obtained in terms of HL among diabetics living in Singapore, where as many as 80.5% presented a high level of health literacy [31]. Differences in results for particular countries may be due to the lack of uniformity in the use of research tools, but also to the socio-demographic characteristics of the study participants and their general health status [32].

In this study, the subject of analysis, apart from the HL level of patients with diabetes and their relationship with demographic features and health status parameters, was also the assessment of their cognitive functions. The study using the MMSE test showed that 33.0% of the patients with diabetes had cognitive disorders of varying intensity. This problem concerned 34.10% of patients with type 1 diabetes and 32.30% with type 2 diabetes, which is consistent with the results of other authors [33].

In this study, the mean test result was 27.12 and was almost identical for patients with type 1 and type 2 diabetes. A statistically significant relationship was found between the age of the patients and the MMSE test result (*p* < 0.001). It was found that the higher the age of the patients, the higher the share of patients with cognitive impairment or dementia. In the studies by Malik et al., the mean MMSE result was lower and amounted to 22.69 ± 5.26, which indicates a higher share of people with cognitive impairment [34], but it is worth noting that it concerned only patients with type 2 diabetes, whose average age was 65.32 (SD = 11.33 years), while the average age of patients in this study was lower and amounted to 54.36 (SD = 15.89 years), which is not without a significance for the risk of cognitive impairment.

Age as a risk factor for the development of cognitive impairment in diabetics has also been demonstrated in other studies [35,36,37], which may additionally coincide with vascular dementia independent of diabetes and other disorders leading to a decline in cognitive functions in elderly patients. Based on the analysis of Spearman’s rho correlation in this study, a statistically significant relationship was shown between the level of education of the examined patients and the result of the MMSE test (*p* < 0.001). A lower level of education turned out to be a predisposing factor to the occurrence of cognitive disorders or dementia in patients, which was confirmed in the study by other authors [38]. However, no significant difference in cognitive impairment was found between women and men, similarly to the study by Malik et al. [34], despite other studies showed that women are at significantly greater risk of developing cognitive dysfunctions [35,39,40].

Moreover, statistical analyses showed only a significant relationship between the length of diabetes (in years) of the examined patients and the general health literacy index, the health care index and the health promotion index. It was found that the higher the number of years of diabetes, the higher the indicators of the discussed tool and its subscales. It should be assumed that this is related to the diabetes education of patients covered by care, which is implemented in Poland. Studies conducted among Polish diabetic patients showed that they have a fairly good state of knowledge about diabetes [41].

Unfortunately, no statistically significant correlation was found between the duration of the disease in the studied patients, the prevention of disease index, the MMSE test result, the occurrence of diabetes complications, place of residence, age, and individual dimensions of general health literacy. No correlation was found between the level of education of the studied patients and individual dimensions of general health literacy either. In the study by Finbråten et al., no correlation was found between the age of the participants and the level of HL; however, a higher level of HL corresponded to a higher level of education (university level) and good general health [27]. The relationship between a higher level of education and a higher level of HL was also confirmed in other studies [42,43,44], but it should be emphasized that all the cited studies concerned only the patients with type 2 diabetes.

The analysis showed that despite the dominant share of diabetic patients included in the study having a sufficient level of HL, almost 40% of the respondents had deficits in this respect. This indicates the need to undertake educational activities to strengthen the general level of HL, but also educational activities adequate for this group of recipients and their families, which could reduce the risk of complications. An approach focused not only on the patient, but also on the community in the field of HL intervention is treated as a chance to achieve better therapeutic results among people with diabetes [45]. In relation to the patient, the course of the educational process is conditioned by many factors. A particularly important role is attributed to psychological factors, but also to the social characteristics and economic situation of the patients, as well as the person of the educator [46]. Equally important seems to be a personalized assessment of the patient’s knowledge and skills, which is a condition for preparing the patient for self-care and self-control. Its scope includes, among others, simplifying therapy schemes, educating the patient and his/her family, and psychotherapy [47].

The benefits of diabetes education are mainly observed in the area of patient self-care, metabolic control of diabetes, adherence to medical recommendations, and the ability to solve problems related to diabetes self-care. Education is also an important element in reducing the burden on healthcare systems [48,49,50].

The main limitations of this study are related to the problematic comparison of the results presented herein with the results of other studies conducted using not only the HLS-EU-47, but also the HLS Q16 and HLS Q12, which provided different final assessments of the obtained results. Additionally, in some studies using the HLS-EU-47, the points were divided into four categories (insufficient, problematic, sufficient, and excellent), while in others, the assessment was divided into three categories (insufficient, problematic and sufficient), providing finally different results and interpretations.

## 5. Conclusions

Both EU HLS-47 and MMSE showed that patients with diabetes mostly presented a sufficient level of health literacy, as indicated by the average score of the obtained answers. A limited level of health literacy was presented by 36.86% of the examined patients with diabetes, with the highest percentage of the examined people with this note referring to the area of health promotion. We found a statistically significant relationship between the length of diabetes (in years) of the examined patients and the general health literacy index, the health care index and the health promotion index. Thus, the greater the number of years of diabetes, the higher the indicators of the EU HLS-47 and its subscales. The MMSE test showed that every third patient with diabetes had cognitive disorders of varying severity. A statistically significant relationship was shown between the age of the examined patients and the result of the MMSE test, the higher the age of the respondents, the higher the share of patients with cognitive disorders or dementia.

Considering the results presented herein, there is a need for educational activities, especially, in the area of health competences related to health prevention and health promotion. Education aimed at eliminating behavioral risk factors of diabetes is recommended, including unhealthy diet, low physical activity, harmful alcohol consumption, and smoking. Education in the field of systematic self-control of glycaemia levels, body weight and the use of recommended pharmacotherapy is highly recommended. Diabetes patients should also be educated about activities that help maintain their mental and social well-being, which will reduce the risk of cognitive and depressive disorders.

## Figures and Tables

**Table 1:** Demographic and social data of patients.

Characteristics of the Studied Patients		N	%
**Gender**	Women	136	43.59
	Men	176	56.41
	Total	312	100.00
**Age**	≥30 years	34	10.90
	31–50	79	25.30
	51–70	162	51.90
	<70	37	11.90
	Total	312	100.00
**Place of residence**	Village	128	41.35
	City	184	58.65
	Total	312	100.00
**Education**	Primary	30	9.60
	Vocational	90	28.80
	Secondary, post-secondary	125	40.10
	Higher	67	21.50
	Total	312	100.00

**Table 2:** Analysis of EU HLS-47 (Polish version).

Question	Load Factor	Mean	SD	Question	Load Factor	Mean	SD	Question	Load Factor	Mean	SD
**Q1.1**	0.46	1.47	0.60	**Q1.17**	0.61	3.43	0.86	**Q1.32**	0.58	3.40	0.79
**Q1.2**	0.64	3.04	0.86	**Q1.18**	0.66	3.04	1.01	**Q1.33**	0.70	3.13	1.00
**Q1.3**	0.67	3.14	0.84	**Q1.19**	0.69	3.13	1.00	**Q1.34**	0.76	3.03	0.98
**Q1.4**	0.68	3.19	0.85	**Q1.20**	0.62	3.31	0.84	**Q1.35**	0.63	2.94	1.15
**Q1.5**	0.73	3.37	0.74	**Q1.21**	0.60	3.51	0.74	**Q1.36**	0.69	3.10	1.09
**Q1.6**	0.71	3.23	0.86	**Q1.22**	0.61	3.38	0.88	**Q1.37**	0.58	3.21	0.91
**Q1.7**	0.71	3.26	0.84	**Q1.23**	0.60	3.29	1.00	**Q1.38**	0.57	3.15	0.98
**Q1.8**	0.72	3.46	0.67	**Q1.24**	0.63	3.47	0.78	**Q1.39**	0.68	3.14	1.00
**Q1.9**	0.63	3.36	0.78	**Q1.25**	0.64	3.29	0.82	**Q1.40**	0.73	3.09	0.95
**Q1.10**	0.58	3.10	0.93	**Q1.26**	0.69	3.10	1.04	**Q1.41**	0.64	3.37	0.85
**Q1.11**	0.61	3.02	1.00	**Q1.27**	0.70	2.96	1.04	**Q1.42**	0.64	3.45	0.78
**Q1.12**	0.47	2.96	1.14	**Q1.28**	0.64	3.01	1.11	**Q1.43**	0.62	3.33	0.85
**Q1.13**	0.56	3.19	0.81	**Q1.29**	0.56	3.13	1.00	**Q1.44**	0.64	3.10	0.95
**Q1.14**	0.66	3.43	0.70	**Q1.30**	0.65	3.03	1.02	**Q1.45**	0.43	3.03	1.07
**Q1.15**	0.56	3.60	0.75	**Q1.31**	0.56	2.97	1.11	**Q1.46**	0.60	3.02	0.98
**Q1.16**	0.56	3.52	0.72	-				**Q1.47**	0.54	2.84	1.08
explained variance 39.27%				explained variance 40.01%				explained variance 39.87%			
Cronbach’s alpha = 0.890				Cronbach’s alpha = 0.891				Cronbach’s alpha = 0.895			

**Table 3:** Age of patients, type of diabetes, gender, and place of residence.

Age	n (%)	Me (Q25–Q75)	Min–Max	*p*-Value *
Total	312 (100)	57 (44–66.5)	18–89	-
Typ II	186 (59.62)	61.5 (54–69)	22–88	*p* < 0.001
Typ I	126 (40.38)	44.5 (32–60)	18–89	
Women	136 (43.59)	59.5 (47–69)	18–88	*p* = 0.024
Men	176 (56.41)	56 (43–64)	18–89	
Village	129 (41.35)	55 (43–65)	19–89	*p* = 0.091
City	183 (58.65)	60 (44–67)	18–88	

* Statistical analysis of data was performed with Mann–Whitney’s U Test.

**Table 4:** The level of health literacy of patients with diabetes.

Question Number from HLS-EU_Q47	Dimension from HLS-EU_Q47	Mean, SD	Categorized Level of Health Literacy	Value of Health Literacy Score (%)
Q1–47	General Health Literacy Index	36.05 ± 8.46	Excellent >42–50	79 (25.32)
			Sufficient >33–42	118 (37.82)
			Problematic >25–33	79 (25.32)
			Inadequate 0–25	36 (11.54)
			Limited (3 + 4) 0–33	115 (36.86)
Q1–16	Health Care	35.62 ± 8.69	Excellent >42–50	79 (25.32)
			Sufficient >33–42	114 (36.54)
			Problematic >25–33	83 (26.60)
			Inadequate 0–25	36 (11.54)
			Limited (3 + 4) 0–33	119 (38.14)
Q17–31	Prevention of Disease	36.57 ± 9.75	Excellent >42–50	94 (30.13)
			Sufficient >33–42	97 (31.09)
			Problematic >25–33	77 (24.68)
			Inadequate 0–25	44 (14.10)
			Limited (3 + 4) 0–33	121 (38.78)
Q32–47	Health Promotion	36.71 ± 9.98	Excellent >42–50	92 (29.49)
			Sufficient >33–42	86 (27.56)
			Problematic >25–33	81 (25.96)
			Inadequate 0–25	53 (16.99)
			Limited (3 + 4) 0–33	134 (42.95)

**Table 5:** Type of diabetes and selected patients’ characteristics.

Variables	Diabetes Type II Me (Q25–Q75)	Diabetes Type I Me (Q25–Q75)	*p*-Value *
Duration of illness (in years)	14.5 (6–20)	16.5 (9–25)	*p* = 0.051
Age (in years)	61.5 (54–69)	44.5 (32–60)	*p* < 0.001
General health literacy index	36 (30–42)	36 (31–42)	*p* = 0.548
Index health care	37 (28–42)	37 (27–41)	*p* = 0.237
Index prevention of disease	37 (30–44)	37 (31–45)	*p* = 0.772
Index health promotion	37 (28–44)	36 (29–43)	*p* = 0.701
MMSE	1 (1–2)	1 (1–2)	*p* = 0.896

* Statistical analysis of data was performed with Mann–Whitney’s U Test.

**Table 6:** Education of patients, age, duration of diabetes, health literacy, and MMSE.

	Education	MMSE	Age	Duration of Diabetes
General health literacy index	−0.023 (*p* = 0.681)	0.026 (*p* = 0.655	−0.010 (*p* = 0.855)	−0.142 (*p* = 0.012)
Index health care	0.009 (*p* = 0.863)	−0.056 (*p* = 0.325)	−0.099 (*p* = 0.080)	−0.148 (*p* = 0.008)
Index prevention of disease	−0.029 (*p* = 0.606)	0.067 (*p* = 0.241)	0.042 (*p* = 0.464)	−0.081 (*p* = 0.155)
Index health promotion	−0.048 (*p* = 0.396)	0.034 (*p* = 0.556)	−0.001 (*p* = 0.980)	−0.164 (*p* = 0.004)
MMSE	−0.340 (*p* < 0.001)	-	0.290 (*p* < 0.001)	0.35 (*p* = 0.542)

Results are presented as the Spearman’s rank-order correlation coefficient (rho) with *p*-values in parentheses.

## Data Availability

The raw data supporting the conclusions of this article will be made available by the authors on request.

## Abbreviations

The following abbreviations are used in this manuscript:

HL	Health Literacy
MMSE	Mini-Mental Scale Examination
EU HLS-47	47-item European Health Literacy Scale
